# Nonhuman Primate Models for HIV Cure Research

**DOI:** 10.1371/journal.ppat.1002892

**Published:** 2012-08-30

**Authors:** Cristian Apetrei, Ivona Pandrea, John W. Mellors

**Affiliations:** 1 Center for Vaccine Research, University of Pittsburgh, Pittsburgh, Pennsylvania, United States of America; 2 Department of Microbiology and Molecular Genetics, School of Medicine, University of Pittsburgh, Pittsburgh, Pennsylvania, United States of America; 3 Department of Pathology, School of Medicine, University of Pittsburgh, Pittsburgh, Pennsylvania, United States of America; 4 Department of Medicine, School of Medicine, University of Pittsburgh, Pittsburgh, Pennsylvania, United States of America; National Institutes of Health, National Institute of Allergy and Infectious Diseases, United States of America

Shytaj et al. [1] report that complete suppression of SIVmac replication can be achieved in rhesus macaques by a combination of five antiretroviral (ARV) drugs, which the authors term “mega-HAART”. This combination consists of the three-drug nucleoside reverse transcriptase inhibitor (tenofovir/emtricitabine) and integrase inhibitor (raltegravir) regimen often used in treatment studies of simian immunodeficiency virus (SIV)-infected rhesus macaques, intensified with the protease inhibitor darunavir (pharmacokinetically enhanced by ritonavir) and the CCR5 antagonist maraviroc. Achieving complete suppression of SIVmac in rhesus macaques is an important step in developing an animal model for HIV-1 cure research because it parallels the effects of antiretroviral therapy in HIV-infected humans. Without complete suppression, testing of therapeutic strategies to reduce viral reservoirs is confounded by ongoing cycles of viral replication that can replete such reservoirs.

Over the last two decades, rhesus macaque models of AIDS have revealed key aspects of HIV-1 pathogenesis, such as virus transmission and early events post-infection, the sites of viral replication and CD4^+^ T cell depletion, and virus and cell turnover [2]–[8]. These models have also been instrumental for vaccine research, allowing the evaluation of increasingly potent DNA and vector immunogens and combinations of these vectors in various prime-boost combinations [9]–[11]. Macaque models of preexposure prophylaxis (PrEP) have also helped elucidate the ARV exposures and the timing of exposure required to maximize protection from virus challenge [12].

By contrast, the rhesus macaque/SIV model has contributed less to the development and optimization of ARV therapy [13]. The main reasons for this include the natural resistance of SIVs to nonnucleoside reverse transcriptase inhibitors (NNRTIs) [14], [15], major differences in ARV pharmacokinetics between humans and macaques [13], [16], and divergent interactions of SIVmac and HIV-1 with host restriction factors [17]. Importantly, because the pandemic HIV-1 subtypes do not replicate in monkey species [18], simian counterparts derived from naturally infected sooty mangabeys, such as SIVmac/smm, must be used [19]. Although SIVsmm is not completely distinct from its HIV relatives, being the cause of the HIV-2 epidemic [20], the differences in ARV susceptibility and pharmacokinetics have restricted the use of the RM/SIVmac models for antiretroviral therapy.

Nevertheless, many ARVs are active in vitro and in vivo against SIVmac [14], [21], and studies of antiretroviral therapy (ART) in macaques have been reported [21]–[24]. In most of these studies, however, complete control of viral replication has not been achieved [14]. In addition to suboptimal pharmacokinetics, failure to achieve complete control of viral replication is likely related to the biology of SIVmac infection in rhesus macaques. SIVmac is more virulent than HIV-1 [3]. The set point of viremia in SIV-infected macaques is 10- to 100-fold higher than in HIV-1 infection, and progression to AIDS occurs in 1–2 years and more quickly (<1 year) in up to 40% of macaques [3], [9]. Alternatives to SIVmac have been reported, most notably the use of chimeric HIV-SIV viruses (called simian-human immunodeficiency viruses or SHIVs) in which SIVmac reverse transcriptase (RT) is replaced by HIV-1 RT (RT-SHIVs) [25]–[27]. RT-SHIVs have the advantage of being susceptible to both nucleoside and non-nucleoside RT inhibitors similar to HIV-1. These chimeric viruses also have limitations, most notably that, similar to the parental virus, RT-SHIVmac is difficult to suppress with the same three-drug combination (tenofovir/emtricitabine/efavirenz) that is most commonly used in humans [25]. As an alternative approach, RT-SHIVmne was constructed using the SIVmne isolate from pigtailed macaques and is used to infect this species. RT-SHIVmne is less virulent than SIVmac and not infrequently can be controlled by the host without intervention. Even so, ART with tenofovir/emtricitabline/efavirenz failed to completely control RT-SHIVmne replication in chronically infected pigtailed macaques, as evidenced by both persistent viral replication and sequence evolution under treatment [26], [27].

The recent report that one patient (“the Berlin patient”) was cured of HIV infection [28] has renewed enthusiasm for a “cure research” aimed at understanding the mechanisms of HIV-1 persistence and developing therapeutic strategies to reduce and ultimately eliminate viral reservoirs. Limitations of human clinical studies, especially invasive sampling of multiple reservoir sites, make it imperative to develop analogous and tractable animal models for cure research. Several groups are trying to achieve this goal by (i) ARV intensification in SIVmac-infected rhesus macaques to completely suppress viral replication, similar to Shytaj et al. [1] (J. Lifson, unpublished data; P. Luciw, unpublished data), (ii) use of Chinese macaques infected with SIVmac (B. Ling, unpublished data), in which viral replication at set point viral replication is lower than in Indian rhesus macaques [29], which have dominated AIDS research, and (iii) development of an animal model of functional cure in the absence of ART [30]. Additionally, humanized mouse models of HIV-1 infection have been developed for cure research [31].

How these varied animal models should be used for cure research is not well defined. Several different therapies could have contributed to the cure of the Berlin patient, including myeloablative chemotherapy, total body irradiation, transplantation of allogeneic Δ32/Δ32stem cells, immunosuppressive agents, and graft-versus-host disease, for which animal models are impractical [28]. Irrespective of the mechanisms of the cure, careful characterization of viral reservoirs is the keystone of cure research. Such studies should involve invasive sampling and detailed description of the sites of virus persistence on completely suppressive ART, as well as identifying the source of viral rebound after cessation of ART. New therapeutic strategies can then be tested, including selective activators of viral expression, reversal of immune exhaustion, and enhancement of viral specific immune responses, with careful quantification of effects on viral reservoirs in different anatomic sites, including the brain. For obvious ethical reasons, such studies cannot be performed in humans. In addition, because the time of infection and the virus inocula can be controlled in animal models, studies can be performed to assess the optimal timing of cure interventions as has been reported for PrEP [12]. In addition, animal models for cure research can establish “proof of concept” for the many new therapeutic strategies emerging in the field before testing in humans. It is essential, however, that the therapeutic benefits observed in animal models are validated in human studies (and vice versa), and that the cause of discrepancies be elucidated. Because none of the current animal models perfectly reproduce HIV-1 infection and ART, it is likely that several different models will be needed to understand virus persistence, latency, reactivation, and eradication.

As such, establishing a nonhuman primate model of complete viral suppression as reported by Shytaj et al. [1] is a step forward for cure research. One may argue that achieving complete control of viral replication is just a baby step towards the overall goal of virus eradication and that much more needs to be accomplished with regard to developing more sensitive virological assays [32], small molecules to activate latent virus [33]–[36], and biologics to clear viral reservoirs [37], [38]. Nevertheless, approaches like that of Shytaj et al. [1] indicate that control of viral replication *is* possible in macaques if drug potent, multidrug combinations are employed and that nonhuman primate models for cure research are here to stay.
